# Butyrate Reduces HFD-Induced Adipocyte Hypertrophy and Metabolic Risk Factors in Obese LDLr-/-.Leiden Mice

**DOI:** 10.3390/nu9070714

**Published:** 2017-07-07

**Authors:** Charlotte E. Pelgrim, Bart A. A. Franx, Jessica Snabel, Robert Kleemann, Ilse A. C. Arnoldussen, Amanda J. Kiliaan

**Affiliations:** 1 Donders Institute for Brain, Cognition and Behaviour, Preclinical Imaging Centre, Department of Anatomy, Radboud University Medical Center, 6525 EZ Nijmegen, The Netherlands; charlottepelgrim@gmail.com (C.E.P.); bart.franx@gmail.com (B.A.A.F.); ilse.arnoldussen@radboudumc.nl (I.A.C.A.); 2 Department of Metabolic Health Research, Netherlands Organisation for Applied Scientific Research (TNO), 2301 CE Leiden, The Netherlands; jessica.snabel@tno.nl (J.S.); robert.kleemann@tno.nl (R.K.)

**Keywords:** obesity, T2DM, high-fat diet, butyrate, adipose tissue, macrophages, adipokines

## Abstract

Adipose tissue (AT) has a modulating role in obesity-induced metabolic complications like type 2 diabetes mellitus (T2DM) via the production of so-called adipokines such as leptin, adiponectin, and resistin. The adipokines are believed to influence other tissues and to affect insulin resistance, liver function, and to increase the risk of T2DM. In this study, we examined the impact of intervention with the short-chain fatty acid butyrate following a high-fat diet (HFD) on AT function and other metabolic risk factors associated with obesity and T2DM in mice during mid- and late life. In both mid- and late adulthood, butyrate reduced HFD-induced adipocyte hypertrophy and elevations in leptin levels, which were associated with body weight, and cholesterol and triglyceride levels. HFD feeding stimulated macrophage accumulation primarily in epididymal AT in both mid- and late life adult mice, which correlated with liver inflammation in late adulthood. In late-adult mice, butyrate diminished increased insulin levels, which were related to adipocyte size and macrophage content in epididymal AT. These results suggest that dietary butyrate supplementation is able to counteract HFD-induced detrimental changes in AT function and metabolic outcomes in late life. These changes underlie the obesity-induced elevated risk of T2DM, and therefore it is suggested that butyrate has potential to attenuate risk factors associated with obesity and T2DM.

## 1. Introduction

In line with the rise of type 2 diabetes mellitus (T2DM) the global prevalence of obesity has more than doubled since 1980 [1]. Moreover, obesity is a major risk factor for T2DM, accounting for 80–85% of the overall risk [2]. Weight or fat loss has the strongest effect on reducing the risk of developing T2DM [3]. It has been suggested that the relationship between obesity and T2DM exists already at a young age [4,5,6]. In addition, obesity is suggested to exaggerate aging processes [7], and aging is associated with impaired insulin signaling [8], which causes an increase in blood glucose levels. Over time, high blood glucose concentrations combined with high blood pressure and high cholesterol levels affect vascular function, and may evolve into cardiovascular disease, kidney failure, and subsequent cognitive deficits [9,10]. 

Accumulating evidence suggests that adipose tissue dysfunction—with adipocyte hypertrophy as a first manifestation—may play a role in the development of obesity-induced metabolic conditions that increase the risk for T2DM [11]. Adipocyte hypertrophy results in an elevated recruitment of macrophages surrounding dead or dying adipocytes, which is referred to as crown-like structures (CLS)—an important hallmark of adipose tissue dysfunction— [12]. Macrophages are believed to be major sources of inflammatory cytokines with detrimental effects on insulin signaling in obesity [13]. In addition, adipocyte hypertrophy may lead to the altered production and secretion of adipokines like leptin, resistin, serum amyloid A (SAA), and adiponectin [14,15,16]. These adipokines play important roles in insulin signaling and the development of a chronic low-grade inflammatory state, which is characteristic for obesity. Overall, amelioration of these obesity-induced and adipose tissue-related changes may provide an important approach to attenuate the risk of developing T2DM. 

There is a growing interest in short-chain fatty acids—such as butyrate—as dietary intervention for obesity and related metabolic diseases [17,18]. Butyrate is one of the fatty acids produced during bacterial fermentation in the distal gut of nondigestible carbohydrates like dietary fiber and resistant starch. Both intake of butyrate and dietary fibers have shown positive effects on body weight and insulin sensitivity [17,19,20,21,22]. In addition, butyrate has anti-inflammatory properties and affects lipogenesis [17]. A decreased concentration of butyrate-producing bacteria has been observed both in patients with obesity [23] and T2DM [24]. Butyrate may therefore play an essential role in the maintenance of an optimal metabolic state.

It is acknowledged that obesity may differentially affect various organs and tissues, like the brain and cardiovascular system, during midlife as opposed to late life [18,25,26,27]. Recently, we demonstrated that butyrate is able to restore high-fat diet (HFD)-induced neuroinflammation and changes in brain function in low-density-lipoprotein receptor knockout (LDLr-/-).Leiden mice in mid-adulthood [18]. In addition, mid-adult mice seem to be more susceptible to the development of HFD-induced cardiovascular damage as compared to late-adult mice [18]. However, it has not been elucidated whether diet-induced obesity affects adipose tissue function and its metabolic outcomes differentially during mid- and late adulthood. As follow-up of the previous published study [18], we investigated in the current study the effects of a therapeutic butyrate intervention on adipose tissue function and adipokine levels in the context of manifested HFD-induced obesity in both mid- and late-adult LDLr-/-.Leiden mice. 

## 2. Materials and Methods 

### 2.1. Animals and Diets

The adipose tissues and plasma samples analyzed in this experiment originate from a previously described study [18]. LDLr-/-.Leiden mice were selected because of their high sensitivity to developing HFD-induced obesity and subsequent complications like vascular damage and fatty liver [28,29,30]. Briefly, male LDLr-/-.Leiden mice (TNO Metabolic Health Research, Leiden, The Netherlands) were housed in individually ventilated cages in a conventional animal room situated in the preclinical imaging center (PRIME) at the central animal laboratory, Radboudumc Nijmegen, the Netherlands. A maximum of five mice were housed per cage with ad libitum access to acidified tap water and food (temperature 21 °C; relative humidity 50–60%; light-dark cycle 7 a.m.–7 p.m.). The experiments were approved by an independent ethical committee on animal care and experimentation (Zeist, The Netherlands), approval project number DEC3682 [18] and carried out in accordance to the ARRIVE guidelines [31]. 

**Figure 1:**
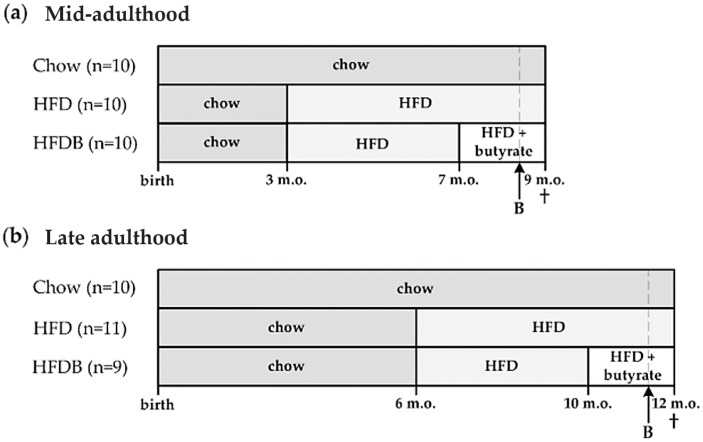
Schematic overview of the study design. (**a**) Mid-adulthood, exposure of the high-fat died HFD started at 3 m.o. in the HFD and HFDB groups. At 7 months of age, the butyrate intervention (HFD supplemented with 5% *w*/*w* butyrate) started in the HFDB group; (**b**) Late adulthood, exposure to the HFD started at 6 months of age in the HFD and HFDB groups, and a butyrate intervention initiated at 10 months of age in the HFDB group. Body weight (individual) and food intake (cage level) were monitored weekly. Blood samples were taken after five hours of fasting. At 9 m.o. and 12 m.o., mice were sacrificed and both liver and adipose tissues were harvested. These tissues were subsequently processed for immunohistochemical staining. B = blood sample collection; HFD = high-fat diet; HFDB = high fat diet enriched with butyrate; m.o. = months old.

The intervention regimen of the study [18] is schematically presented in Figure 1. A total group of *n* = 60 LDLr-/-.Leiden mice were divided into a mid- and late-adult cohort (*n* = 30 each). Both cohorts consisted of three diet groups. For the cohort representing mid-adulthood, the first group received a chow diet from birth until the end of the study (Chow). The second group switched to a HFD at three months of age (HFD). The third group switched to a HFD when three months old (m.o.) as well, but this HFD was subsequently enriched with 5% *w*/*w* butyrate from seven months of age until the end of the study (HDFB) (Figure 1a). The cohort representing late adulthood was subdivided in the same manner, but mice were switched to HFD at six months of age and butyrate intervention started at 10 months of age (Figure 1b). 

### 2.2. Plasma Analyses

Blood was collected after five hours of fasting (8 a.m. to 1 p.m.). Plasma cholesterol, triglyceride and insulin levels were measured using standardized ELISA kits as previously described [18]. Moreover, ELISA kits (Quantikine, R&D systems, Inc., Minneapolis, MN, USA) were used to define plasma leptin (DY498), adiponectin (DY1119), resistin (DY1069) and interleukin-6 (IL-6; M6000B) levels. SAA plasma levels were determined by an ELISA kit as well (KMA0021, Invitrogen, Carlsbad, CA, USA). 

### 2.3. (Immuno)histochemistry

Mice were sacrificed by transcardial perfusion with 0.1 M phosphate buffered saline (PBS) after being anesthetized with isoflurane (3.5%, Nicholas Primal (I) limited, London, UK), as described in Arnoldussen et al. [18]. Directly thereafter, the liver and two different adipose tissue depots were harvested and post-fixed in 4% paraformaldehyde for 24 h. Fat depots seem to be differentially active and affected by a HFD and, therefore, we dissected in addition subcutaneous (inguinal) and visceral (epididymal) adipose tissue depots [32,33]. Liver outcomes used for correlation analyses were obtained from a previous experiment [18]. 

Both epididymal and inguinal adipose tissues were processed for immunohistochemistry on 5 μm paraffin embedded sections. Briefly, paraffin sections were first deparaffinized in xylene and rehydrated in a series of ethanol followed by endogenous peroxidase blocking with 0.3% H_2_O_2_ in 0.1 M PBS. Antigen retrieval was achieved by treating the tissue in hot 0.05 M sodiumcitrate with a constant temperature of 85 °C. After pre-incubation with 0.1% bovine serum albumin in 0.1 M PBS (PBS-B), the tissue was incubated overnight at room temperature with primary macrophage (MAC-3) antibodies (1:100; eBioscience, 14-1072) [34]. Next, sections were incubated with secondary donkey anti-rat IgG antibodies (1:200; Jackson ImmunoResearch) in 0.1 M PBS-B at room temperature. Staining was visualized using the ABC method with a Vectastain kit (Vector Laboratories, Burlingame, CA, USA) and diaminobenzidine imidazole solution as chromogen. Finally, sections were dehydrated in a series of ethanol, cleared in xylene, and mounted. 

Two entire sections per animal were visualized using a VisionTek live digital microscope (Sakura Finetek, Torrance, CA, USA) at a 10× magnification. Sampling of these sections was carried out with Adobe Illustrator CC 2016 (Adobe, San Jose, CA, USA) using a template with three randomly placed artboards (592.7 × 886.2 mm). Adipocyte cell size (μm^2^) and the number of CLS were determined in a total of six samples—three samples per section—per animal. Analyses to determine these measures were performed using CellProfiler [35] version 2.3.0 together with Ilastik version 0.5 [36] for pixel classification. Separate pipelines and classifiers for epididymal and inguinal adipose tissue analyses were used. In order to verify the reliability of these relatively novel analyses, we included an established analysis to determine adipocyte size using ImageJ software as previously described [37]. CLS were determined based on intensity of the cell edge, and total numbers of CLS per animal were calculated per 1000 adipocytes. 

### 2.4. Statistical Analyses

A random and blinded selection procedure was maintained throughout the study. Group means were compared using univariate analysis of variance (ANOVA) with Bonferroni correction for multiple testing with a statistical program, SPSS 24 (IBM SPSS Statistics 24, IBM Corporation, Armonk, NY, USA). Parameter correlation tests were performed using Pearson correlations. Nonparametric tests were used when assumptions of normality and homogeneity of variance were not met. *p*-values lower than 0.05 were considered significant. Data are presented as mean ± SEM. 

## 3. Results

### 3.1. Adipocyte Size

**Figure 2:**
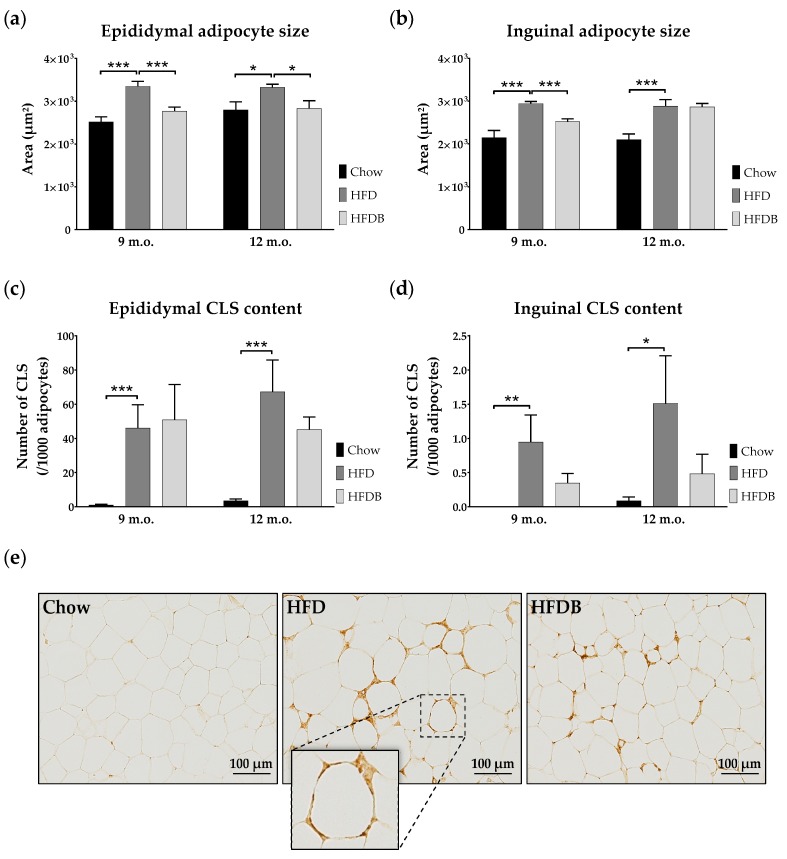
Adipose tissue cell sizes and macrophage infiltration. Mean adipocyte sizes in epididymal (**a**) and inguinal (**b**) adipose tissue depots in both mid- (9 m.o.) and late- (12 m.o.) adult mice. (**c**,**d**) Mean number of crown-like structures (CLS) in epididymal (**c**) and inguinal (**d**) adipose tissue. (**e**) Representative photomicrographs of epididymal adipose tissue stained with antibodies against macrophages (MAC-3) in late-adult mice. In addition, a magnification (40×) of a CLS is represented. These examples are comparable to those observed in mid-adulthood. Data are presented as mean ± standard error of mean (SEM). * *p* < 0.05; ** *p* < 0.01; *** *p* ≤ 0.001.

Adipocyte size—measured as area (μm^2^)—of both epididymal and inguinal adipose tissue depots were determined to assess morphological adaptations. A HFD increased the average epididymal adipocyte size in both mid- and late-adult mice (mid: *p* < 0.001; *F*(1,18) = 26.05, late: *p* < 0.02; *F*(1,19) = 7.01) (Figure 2a). Butyrate intervention significantly reduced adipocyte size in both age cohorts (mid: *p* = 0.001; *F*(1,18) = 14.95, late: *p* < 0.02; *F*(1,18) = 7.14). 

Similarly, inguinal adipocyte size was increased by HFD in both mid- and late adulthood (*p* = 0.001; mid: *F*(1,18) = 20.49, late: *F*(1,19) = 14.25) (Figure 2b). After HFD exposure, inguinal adipocyte size was reduced due to butyrate intervention in mid-adult mice (*p* < 0.001; *F*(1,18) = 25.69), but not in late-adult mice. Mean inguinal adipocyte size was significantly larger in late adult as compared to mid-adult high fat diet enriched with butyrate (HFDB) mice (*p* < 0.01; *F*(1,18) = 11.28). 

In addition, we compared the number of adipocytes as an additional indication of adipose tissue function. Our analysis indicated that the epididymal adipose tissue contained a lower number of adipocytes than inguinal adipose tissue. The mean adipocyte number in epididymal adipose tissue was higher in mid-adult Chow mice as compared to HFD mice (Table 1). Butyrate intervention increased epididymal adipocyte number solely in mid-adulthood. In both age cohorts, inguinal adipocyte numbers were significantly higher in Chow mice as opposed to HFD mice (Table 1). Only in mid-adulthood did butyrate intervention increase the inguinal adipocyte numbers. 

In mid- and late adulthood, both epididymal and inguinal adipocyte size were positively associated with body weight, and cholesterol and triglyceride plasma levels (Table 2 and Table 3). In addition, only inguinal adipocyte size was positively correlated with inguinal adipose tissue weight (mid: *p* < 0.01; *R* = 0.55, late: *p* < 0.001; *R* = 0.64). Solely in late adulthood, epididymal adipocyte size correlated positively with plasma insulin levels (*p* < 0.01; Table 3). 

Moreover, body weight, cholesterol and triglyceride levels revealed significant and positive inter correlations (Table 2 and Table 3). Finally, insulin levels correlated positively with these outcomes in late-adult mice (Table 3).

### 3.2. Inflammation in Adipose Tissue and Liver

To assess the degree of inflammation in adipose tissue, the number of adipocytes infiltrated by macrophages—known as crown-like structures (CLS)—has been determined. Both mid- and late-adult mice on HFD showed higher numbers of CLS in epididymal adipose tissue as opposed to Chow-fed mice (Kruskal Wallis: *p* < 0.001; mid: χ^2^(1) = 13.50, late: χ^2^(1) = 13.02) (Figure 2c). No effects of butyrate on CLS number in epididymal adipose tissue were observed. In inguinal adipose tissue, the number of CLS was increased after HFD exposure in both mid- (Kruskal-Wallis: *p* < 0.01; χ^2^(1) = 7.30) and late- (Kruskal-Wallis: *p* < 0.02; χ^2^(1) = 6.16) adult mice (Figure 2d). No significant differences in the number of CLS in inguinal adipose tissue were observed between HFD and HFDB mice (Figure 2d). As scales differ between Figure 2c,d, caution should be taken when interpreting these results. In Figure 2e, examples of epididymal adipose tissue stained for macrophages are shown.

In late adulthood, the number of CLS in epididymal adipose tissue correlated significantly with body weight (*p* = 0.001), and plasma triglyceride (*p* < 0.02), cholesterol (*p* < 0.001) and insulin (*p* < 0.05) levels (Table 3). In mid-adulthood, epididymal adipose tissue CLS content showed only significant correlations with body weight (*p* < 0.001) and plasma cholesterol levels (*p* < 0.001) (Table 2). 

In addition to CLS in adipose tissue, data including inflammatory aggregates were used [18] to examine potential relationships to inflammation in the liver, which is another crucial organ regulating insulin sensitivity. Liver inflammation was increased after HFD exposure only in late-adult mice [18]. No significant changes were observed after butyrate intervention. In this age cohort, the number of inflammatory aggregates in the liver was positively correlated with the number of CLS in epididymal adipose tissue (*p* < 0.001) (Table 3). 

### 3.3. Adipokine Plasma Levels

**Figure 3:**
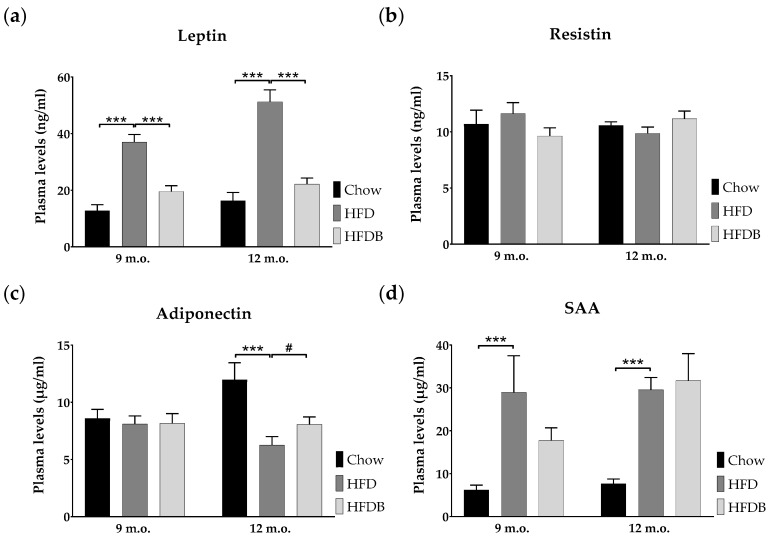
Adipokine plasma levels. Mean plasma levels of leptin (**a**), resistin (**b**), adiponectin (**c**), and serum amyloid A (SAA) (**d**) are represented in both mid- (9 m.o.) and late- (12 m.o.) adult mice as mean ± SEM. *** *p* ≤ 0.001; # *p* = 0.053.

Adipokine plasma levels were determined at either nine or 12 months of age. HFD exposure strongly increased leptin plasma levels in both age cohorts (*p* < 0.001; mid: *F*(1,18) = 50.10, late: *F*(1,19) = 44.87) (Figure 3a). Late-adult HFD-fed mice showed higher leptin levels as compared to mid-adult mice fed a HFD (*p* < 0.02; *F*(1,19) = 7.71). Subsequent butyrate intervention resulted in reduced plasma leptin levels, almost reaching control levels in Chow mice (*p* < 0.001; mid: *F*(1,18) = 26.49, late: *F*(1,18) = 32.88). HFD did not affect plasma resistin levels (Figure 3b). Adiponectin plasma levels were reduced after HFD exposure in late-adult mice (*p* < 0.001; *F*(1,19) = 17.57) and were moderately increased after butyrate intervention (*p* = 0.053; *F*(1,18) = 4.29) (Figure 3c). An age effect was observed in both Chow and HFD animals (*p* < 0.05; *F*(1,18) = 4.60 and *F*(1,19) = 4.55, respectively). Serum amyloid A (SAA) plasma levels were increased in mice fed a HFD when compared to Chow-fed mice in both age cohorts (Kruskal-Wallis: *p* ≤ 0.001; mid: χ^2^(1) = 10.14, late: χ^2^(1) = 14.46) (Figure 3d). Plasma levels of interleukin-6 (IL-6) were below detectable levels in all animals in the absence of abnormal positive and negative controls. 

In both mid- and late adulthood, plasma leptin levels were positively correlated with body weight (*p* < 0.001), both epididymal and inguinal adipocyte size (*p* < 0.001), and epididymal CLS content (*p* < 0.001) (Table 2 and Table 3). Solely in late-adult mice, plasma leptin levels positively correlated with insulin levels (*p* < 0.001) and liver inflammation (*p* = 0.001) (Table 3). Only in late adulthood plasma adiponectin levels showed a negative correlation with body weight (*p* < 0.01), insulin plasma levels (*p* < 0.02), and liver inflammation (*p* < 0.05) (Table 3). 

Plasma SAA levels were positively correlated with body weight (mid: *p* < 0.001, late: *p* < 0.01) and the number of CLS in epididymal adipose tissue (*p* < 0.001) in both mid- and late adulthood (Table 2 and Table 3). Whereas, SAA plasma levels were positively correlated with insulin levels (*p* < 0.05), inguinal adipocyte size (*p* < 0.001), and liver inflammation (*p* < 0.01) only in late adulthood (Table 3).

## 4. Discussion

This study describes the detrimental effects of a six-month HFD exposure in mid-adult and late-adult mice, and examined potential health effects of butyrate administered during the last two months of HFD feeding. In this study, HFD exposure caused adipocyte hypertrophy and inflammation, as well as an increase in the plasma levels of proinflammatory adipokines (leptin, SAA) in mid- and late life. Butyrate attenuated HFD-evoked effects, though the effects of butyrate varied in mice that were exposed to HFD in mid-life as compared to late life. 

In our study, HFD treatment increased body weight and adiposity, and subsequent butyrate intervention diminished these changes in the LDLr-/-.Leiden mice [18]. Moreover, these changes were observed without significant differences in calorie intake, suggesting that energy intake was similar between the dietary interventions. Other rodent research has shown that a HFD increases body weight and adiposity [38,39,40], and similar effects of butyrate have been reported in obese mice [38,41]. The LDLr-/-.Leiden mouse model was chosen for this study because of its high sensitivity to develop vascular complications and obesity when fed a HFD even with modest fat content. We found that 12 m.o. LDLr-/-.Leiden mice on standard Chow show a disposition towards pronounced weight gain and are approximately 32% heavier than Chow-fed male C57BL/6 mice of the same age [42], supporting the obesity-prone condition of the model chosen. It is unclear why these mice are susceptible to obesity development, and comparative molecular analysis to C57BL/6 mice have not been made. In our experimental design, a control group of wild-type mice was not included due to restrictions in mice per experiment and time. Therefore, a generalization of the results cannot be made, the more so because the interaction between the LDLr-/- genotype and HFD are still not fully understood. A detailed comparison with wild-type mice and other obesity models would be required to elucidate the role of the LDLr in obesity. 

In a previous study, insulin levels were significantly increased in late-adult mice after HFD feeding, whereas no changes in glucose levels were observed [18]. Plasma insulin levels are extremely high due to the experimental conditions employed in this disease model (>20 ng/mL) and much higher than in wild-type mice fed the same diet (<5 ng/mL) [18,30], indicating insulin resistance but no overt T2DM because glucose levels are not elevated and seem to be controlled. Interestingly, butyrate supplementation restored insulin levels to values found in Chow-fed mice, suggesting an improvement in insulin signaling [18]. Previous research described similar effects of butyrate on insulin levels and sensitivity in HFD-fed mice [38,43]. It is not clear whether this effect of butyrate is mediated by improvement of insulin signaling in liver, in peripheral organs, or both. It is possible that both organs are involved, given the time frame in which insulin resistance develops under the conditions employed. For instance, a time resolved clamp study using C57BL/6 mice that were treated with the same HFD as used in the current study showed that the adipose tissue develops insulin resistance after about six to 12 weeks of HFD feeding [44]. Second, the development of insulin resistance in the liver requires longer periods of HFD feeding (about 24 weeks), i.e., a period that is comparable to the duration of HFD exposure herein [44]. 

Even in the absence of obesity, metabolic dysfunction in adipose tissue induced by adipocyte hypertrophy has consistently been related to insulin resistance and may serve as a predictor for T2DM [45,46]. We observed increased adipocyte size in both epididymal and inguinal adipose tissue depots after HFD feeding. Adipocyte hypertrophy was reduced by butyrate with the exception of inguinal adipose tissue in late-adult mice. Epididymal adipocyte sizes were associated with insulin levels in late adulthood suggesting that epididymal, as opposed to inguinal, adipose tissue may be important in mediating the effects on insulin. Previous studies have demonstrated the significant role of visceral fat in mediating insulin sensitivity in rats [47,48], which may be attributed to the higher degree of metabolic activity of visceral fat as opposed to subcutaneous fat [16,49,50]. The beneficial effects of butyrate on adipocyte size in epididymal adipose tissue have been demonstrated before [51]. Although our knowledge about the exact mechanisms is incomplete, it is likely that butyrate may reverse adipocyte hypertrophy by promoting fatty acid oxidation in adipose tissue [38]. In particular, butyrate may induce a shift from lipid synthesis to utilization resulting in decreased fat storage in white adipose tissue [43]. Our data confirm this hypothesis as adipocyte sizes were significantly associated with both cholesterol and triglyceride levels. Dyslipidemia is a regularly observed condition in T2DM patients [52]. HFD-induced elevations in cholesterol and triglycerides were diminished by butyrate in LDLr-/-.Leiden mice [18], suggesting a mitigation of hyperlipidemia that is not related to enhanced clearance, suggesting effects on very-low-density lipoprotein (VLDL) production. For instance, butyrate may affect hyperlipidemia as it can induce a switch from lipid synthesis to utilization [53]. Unfortunately, our study is limited with respect to functional analyses such as glucose transport, insulin signaling, lipogenesis, and adipogenesis in adipose tissue. Therefore, it would be valuable to investigate these aspects in future studies. The effects of butyrate supplementation on key regulators of adipogenesis in mid-adult versus late-adult mice may provide insight into the pathways by which butyrate may modulate adiposity. It has been shown that butyrate may reduce body weight and improve insulin sensitivity via the modulation of peroxisome proliferator-activated receptor-γ (PPAR-γ) [43], which is a key regulator of adipogenesis [54]. Consistent with this, we recently showed that specific activation of PPAR-γ in adipose tissue attenuates CLS development and, related to this, the development of nonalcoholic fatty liver disease/nonalcoholic steatohepatitis [55]. Anti-inflammatory effects in adipose tissue may attenuate the release of inflammatory mediators and may also affect the development of diseases in other organs such as the liver [56,57]. A notion which is further supported by the coordinated and interactive expression of inflammatory genes in adipose tissue and liver during metabolic overload [58].

It is well established that a correlative and causative relation exists between chronic inflammation and both insulin resistance and T2DM in humans and rodents [13,59]. More specifically, a reduction of macrophage infiltration in adipose tissue can improve insulin sensitivity in diet-induced obese mice by reducing the expression of inflammatory cytokines in adipose tissue [11]. As shown in the present study, HFD feeding results in increased macrophage infiltration mainly in epididymal adipose tissue associated with body weight and insulin levels. Epididymal adipose tissue was characterized by an abundance of CLS, whereas CLS were almost absent in inguinal adipose tissue, which is consistent with previous studies investigating adipose tissue inflammation of various adipose tissue depots in the context of their expandability and maximal storage capacity [30,57]. It is possible that visceral fat may excrete higher amounts of inflammatory proteins and fatty acids affecting insulin signaling, and that macrophages play an important role in this [56,60]. CLS formation in epididymal adipose tissue was not affected by butyrate supplementation in mid-adult mice, whereas a mean reduction of almost 33% was observed in late-adult mice. Consistent with the inflammatory cross-talk between epidydimal adipose tissue and liver, medial lobular inflammation was not affected in mid-adult mice, whereas the number of inflammatory aggregates in the liver was reduced by 44% after dietary butyrate intervention in late-adult mice [18]. Furthermore, liver inflammation in late-adult mice, indicated by inflammatory aggregates in the medial lobe [18], correlated significantly with the number of CLS in epididymal adipose tissue in this study. It is not known via which pathways butyrate can affect inflammation, and future studies may examine the expression of inflammatory mediators at tissue level, in both adipose tissue and the liver. In addition, mechanistic studies examining the expression of transcription factors, such as nuclear factor κB, support the notion that the activation of inflammatory processes in adipose tissue and liver are necessary. 

Both the increase in pro-inflammatory and the reduction in anti-inflammatory adipokine secretion by adipose tissue can contribute to systemic and tissue-specific inflammation. In this study, HFD exposure results in increased levels of leptin and serum amyloid A (SAA). These findings are accompanied by a reduction in adiponectin in late adulthood, whereas it remains unclear why adiponectin was not affected by HFD or butyrate treatment in mid-adult mice. An exaggerated metabolic load in adipocytes and inflammatory state may underlie the effects of a HFD on leptin, adiponectin, and SAA levels [16]. Both leptin and SAA are suggested to be pro-inflammatory adipokines, whereas adiponectin has anti-inflammatory properties [61]. Leptin contributes to the inflammatory state in obesity by modulating TNF-α and activating macrophages [62], and butyrate reduced leptin levels in mid-adult and late-adult mice. Leptin can also affect insulin sensitivity by modulating pancreatic β-cells [60]. It thereby contributes to the observed reduction in plasma insulin by butyrate in late-adult mice the more, because butyrate reduced leptin levels nearly to those of Chow-fed mice. Our finding that leptin levels were significantly related to insulin levels in late-adult mice further supports this notion. Although current evidence for the role of SAA in insulin signaling is contradictory [63,64,65], SAA levels have been correlated with insulin resistance and T2DM [66], which may be related to the pro-inflammatory properties of SAA [14]. Interestingly, adiponectin plasma levels were only affected by HFD feeding in late-adult mice. It is possible that the circulating SAA is mainly released from adipose tissue and that butyrate reduced CLS only in adipose tissue of late-adult mice. Low adiponectin levels may add to an impaired insulin signaling via polarization towards M1 macrophages in adipose tissue [67]. Butyrate intervention reduced changes in both leptin and adiponectin levels, which may in part be attributed to its effect on adipocyte size and its differential effects on CLS, i.e., adipose tissue inflammation, in mid-and late-adult mice. 

## 5. Conclusions

This study provides evidence that dietary supplementation with butyrate constitutes a strategy to counteract features of HFD-induced adipose tissue dysfunction and accompanying metabolic disturbances. These metabolic adaptations often affect insulin signaling and may therefore indicate an increased risk of developing T2DM. Late-adult mice are particularly sensitive to HFD-evoked metabolic risk factors and adaptations. The findings of the current study provide novel insights about the attenuating effects of butyrate on adipose tissue dysfunction and inflammation. Overall, dietary interventions with butyrate may constitute means to ameliorate the risk of developing metabolic disorders such as T2DM in people with obesity. 

## Figures and Tables

**Table 1:** Adipocyte numbers. Represented are the combined mean numbers of two slides.

Adipocyte Numbers Per 10^6^ μm^2^ (Mean ± SEM)				
**Epididymal**				
**Chow vs. HFD**	mid	468 ± 34 vs. 376 ± 31	0.05 < *p* < 0.08	*F*(1,18) = 3.95
	late	452 ± 40 vs. 410 ± 21	*p* = 0.02	*F*(1,18) = 6.54
**HFD vs. HFDB**	mid	376 ± 31 vs. 496 ± 35	ns	
	late	410 ± 21 vs. 481 ± 47	ns	
**Inguinal**				
**Chow vs. HFD**	mid	665 ± 59 vs. 449 ± 16	*p* < 0.01	*F*(1,18) = 12.61
	late	659 ± 44 vs. 474 ± 41	*p* < 0.01	*F*(1,19) = 9.54
**HFD vs. HFDB**	mid	449 ± 16 vs. 522 ± 15	*p* < 0.01	*F*(1,18) = 11.27
	late	474 ± 41 vs. 446 ± 18	ns	

ns = not significant.

**Table 2:** Correlation coefficients among mid-adult mice.

	BW		Chol		Trigl		eAS		iAS		eCLS		iCLS		Leptin	SAA
BW	x															
Chol	0.80 ^R^	+	x													
Trigl	0.58 ^R^	+	0.69 ^R^	+	x											
eAS	0.53 ^R^	+	0.64 ^R^	+	0.57 ^R^	+	x									
iAS	0.55 ^R^	+	0.57 ^R^	+	0.37 ^R^	+	NE		x							
eCLS	0.53 ^τ^^b^	+	0.49 ^τ^^b^	+	0.15 ^τ^^b^	+	0.24 ^τ^^b^	+	NE		x					
iCLS	0.31 ^τ^^b^	+	0.29 ^τ^^b^	+	0.10 ^τ^^b^	+	NE		0.37 ^τ^^b^	+	NE		x			
Leptin	0.78 ^R^	+	0.87 ^R^	+	0.62 ^R^	+	0.76 ^R^	+	0.67 ^R^	+	0.52 ^τ^^b^	+	0.37 ^τ^^b^	+	x	
SAA	0.56 ^τ^^b^	+	0.50 ^τ^^b^	+	0.13 ^τ^^b^	+	0.17 ^τ^^b^	+	0.24 ^τb^	+	0.69 ^τb^	+	0.28 ^τ^^b^	+	NE	x

Either the Pearson’s (^R^) or Kendall’s tau (^τb^) test was used. Not significant. + = positive correlation; Adi = adiponectin; BW = body weight; Chol = cholesterol; eAS = epididymal adipocyte size; eCLS = epididymal CLS; iAS = inguinal adipocyte size; iCLS = inguinal CLS; LI = liver inflammation; NE = not estimated; SAA = serum amyloid A; Trigl = triglycerides.

**Table 3:** Correlation coefficients among late-adult mice.

	BW		Insulin		Chol		Trigl		eAS		iAS		eCLS		iCLS		LI		Leptin	Adi	SAA
BW	x																				
Insulin	0.66 ^R^	+	x																		
Chol	0.87 ^R^	+	0.66 ^R^	+	x																
Trigl	0.79 ^R^	+	0.77 ^R^	+	0.81 ^R^	+	x														
eAS	0.52 ^R^	+	0.49 ^R^	+	0.57 ^R^	+	0.53 ^R^	+	x												
iAS	0.47 ^R^	+	0.31 ^R^	+	0.54 ^R^	+	0.50 ^R^	+	NE		x										
eCLS	0.43 ^τb^	+	0.30 ^τb^	+	0.55 ^τb^	+	0.34 ^τb^	+	0.23 ^τ^^b^	+	NE		x								
iCLS	0.45 ^τb^	+	0.21 ^τ^^b^	+	0.38 ^τ^^b^	+	0.33 ^τ^^b^	+	NE		0.37 ^τ^^b^	+	NE		x						
LI	0.41 ^τ^^b^	+	0.33 ^τ^^b^	+	0.45 ^τ^^b^	+	0.30 ^τ^^b^	+	0.15 ^τ^^b^	+	0.22 ^τ^^b^	+	0.57 ^τ^^b^	+	0.21 ^τ^^b^	+	x				
Leptin	0.88 ^R^	+	0.69 ^R^	+	0.91 ^R^	+	0.87 ^R^	+	0.65 ^R^	+	0.64 ^R^	+	0.49 ^τb^	+	0.41 ^τb^	+	0.46 ^τb^	+	x		
Adi	0.56 ^R^	−	0.48 ^R^	−	0.68 ^R^	−	0.47 ^R^	−	0.25 ^R^	−	0.25 ^R^	−	0.19 ^τ^^b^	−	0.30 ^τ^^b^	−	0.28 ^τ^^b^	−	NE	x	
SAA	0.42 ^τ^^b^	+	0.29 ^τ^^b^	+	0.57 ^τ^^b^	+	0.46 ^τ^^b^	+	0.19 ^τ^^b^	+	0.53 ^τ^^b^	+	0.58 ^τ^^b^	+	0.30 ^τ^^b^	+	0.44 ^τb^	+	NE	NE	x

Either the Pearson’s (^R^) or Kendall’s tau (^τb^) test was used. Not significant. + = positive correlation; − = negative correlation; Adi = adiponectin; BW = Body weight; Chol = cholesterol; eAS = epididymal adipocyte size; eCLS = epididymal CLS; iAS = inguinal adipocyte size; iCLS = inguinal CLS; LI = liver inflammation; NE = not estimated; SAA = serum amyloid A; Trigl = triglycerides.
